# A biomechanical characterisation of acellular porcine super flexor tendons for use in anterior cruciate ligament replacement: Investigation into the effects of fat reduction and bioburden reduction bioprocesses

**DOI:** 10.1016/j.jbiomech.2014.11.013

**Published:** 2015-01-02

**Authors:** Anthony Herbert, Gemma L. Jones, Eileen Ingham, John Fisher

**Affiliations:** a (IMBE) Institute of Medical and Biological Engineering, School of Mechanical Engineering University of Leeds, Leeds, UK; b IMBE, Faculty of Biomedical Sciences, University of Leeds, Leeds, UK

**Keywords:** Anterior cruciate ligament, Knee reconstruction, Porcine, Decellularisation, Acellular graft

## Abstract

The decellularisation of xenogenic and allogeneic biological grafts offers a promising solution to replacement of the anterior cruciate ligament (ACL). The purpose of this investigation was to determine the biomechanical effects of additional fat reduction and bioburden reduction steps in the decellularisation of porcine super flexor tendon (pSFT). Study 1 investigated the use of acetone or chloroform–methanol as a fat reduction agent. The most effective of these was then carried forward into Study 2, which investigated the use of antibiotics or peracetic acid (PAA) as a bioburden reduction agent. Stress relaxation data was analysed using a Maxwell–Wiechert viscoelastic model and, in addition to classical material properties, the tangent modulus of the toe-region was determined from strength testing data. In both studies, the majority of decellularised groups demonstrated no statistical differences for material properties such as tensile strength and Young’s modulus compared to native controls. Different trends were observed for many of the viscoelastic parameters, but also for the tangent modulus in the toe-region indicating a change in performance at low strains. The most severe deviations from the profile of the native tangent modulus were found to occur in Study 2 when PAA was used for bioburden reduction. Classic material properties (*E*, *UTS* etc.) are often used to compare the characteristics of native and decellularised tissues, however they may not highlight changes occurring in the tissues at low strains. In this study, this represented the physiological strains encountered by substitute acellular ACL grafts. Acetone was chosen as the fat reduction step whereas, antibiotics was preferable over PAA as a bioburden reduction step.

## 1 Introduction

Rupture of the anterior cruciate ligament (ACL) is becoming a more prevalent issue in younger populations with increasingly more active lifestyles. It has been estimated to occur at an annual rate of 1 in 3000 in the US alone, translating to over 100,000 reconstruction surgeries to restore joint stability (Woods and Gratzer, 2005). Damage to the ACL leads to instability of the knee and reduced function, which in turn may lead to damage to the meniscus and degenerative joint disease such as osteoarthritis (Corry et al., 1999; Spindler and Wright, 2008). Surgical intervention is required to restore stability to the knee.

Bone-patellar tendon‐bone autografts present one treatment option but can often induce complications such as knee stiffness, patellofemoral pain and donor site morbidity, symptomatic to the graft’s harvest (Peterson et al., 2001). An alternative treatment choice is reconstruction of the ACL using semitendinosus or gracilis tendon autograft, which has increased in use with significant improvements in fixation methods (Aga et al., 2013; Kousa et al., 2003a, 2003b; Robbe and Paletta, 2004; Samuelsson et al., 2013). However, these grafts also suffer complications of donor site morbidity and nerve damage during the harvesting procedure remains a possibility. Allografts may also be used, but their success may be limited by adverse immunological reactions (Prokopis and Schepsis, 1999). Additionally, it is unlikely that the cells present in allografts and autografts remain viable and this can lead to deterioration of the mechanical properties over time (Woods and Gratzer, 2005). This is because the rate of tissue degradation (as a result of graft necrosis) typically exceeds that of cellular repopulation, collagen remodelling and maturation (Corsetti and Jackson, 1996; Mcfarland, 1993). The decellularisation of allogeneic or xenogenic tissue grafts to remove the cells and immunogenic components offers a promising alternative to current solutions.

Decellularisation techniques have been applied to a number of biological tissues to create acellular biological scaffolds. These include cartilage (Yang et al., 2010), bladder (Bolland et al., 2007), heart valves (Booth et al., 2002; Knight et al., 2008; Korossis et al., 2002), meniscus (Stapleton et al., 2008), in addition to ligaments (Harrison and Gratzer, 2005; Ingram et al., 2007; Tischer et al., 2010; Woods and Gratzer, 2005) and tendon (Pridgen et al., 2011). The ideal acellular biological scaffold should be biocompatible, promote cell in-growth, have the ability to be remodelled and possess the necessary mechanical properties to survive at the site of implantation. The latter becomes an issue of great importance with reference to ACL reconstruction as the acellular biological scaffold would need to maintain joint stability under intensive, repetitive loading conditions. Hence, it is vital that the decellularation process applied to ligament and tendon grafts preserves the graft tissue’s mechanical properties as much as possible in order to maintain sufficient mechanical integrity during the regenerative process in-vivo.

We have identified the porcine super flexor tendon (pSFT) as a possible candidate for use in ACL reconstruction. The pSFT is readily procured and once split along its long axis offers double bundle deployment opportunities similar to hamstring tendon autografts. The aim of this study was to investigate the effects of the decellularisation process and of variations thereupon on the mechanical properties of the pSFT. Initial decellularisation was achieved using an adaptation of a previously used protocol for the meniscus (Stapleton et al., 2008). The subsequent effects of including fat reducing agents (acetone or chloroform–methanol) in the decellularisation process on the biomechanical properties were also determined by comparison to native tissue (Study 1). The superior fat reduction agent was then incorporated into the next generation of the decellularisation process. Following this, a further study (Study 2) investigated the effects of including different sterilisation regimens upon the biomechanical properties of the decellularised tissue. This was performed by including either peracetic acid (PAA) or antibiotics as an initial bioburden reduction step and investigating how their inclusion affected the mechanics with/without PAA as a terminal sterilant.

## 2 Materials and methods

### 2.1 Tissue sourcing

Female ~70 kg, 4 month old, large white pigs were obtained from the abattoir (J. Penny, Leeds, UK) within 24 h of slaughter. Once removed, all pSFT’s were stored at −20 °C with phosphate buffered saline (PBS) soaked filter paper prior to preparation and testing, whether they contributed to native (untreated) or decellularised test groups. A population size of *n*=6 was chosen for all test groups investigated.

### 2.2 Decellularisation

#### 2.2.1 Study 1: Fat reduction study

pSFT’s allocated for decellularisation were subjected to three freeze-thaw cycles. After the first thaw, the pSFT׳s were frozen in hypotonic buffer supplemented with protease inhibitors (10 mM tris, 2.7 mM EDTA [Fisher Scientific], 10 KIU ml^−1^ aprotinin [NHS Supplies, Leeds, UK]). The hypotonic buffer was replenished after the second thaw. For fat reduction, the tendons were then subjected to either 3×1 h washes with acetone (VWR International) at room temperature, or 1×1 h with chloroform–methanol (2:1; v/v [VWR, Atom Scientific]) and 1×30 min wash with methanol (Atom Scientific) at room temperature. Tendons were subsequently washed five times for 5 min in PBS plus 10 KIU ml^−1^ aprotinin (NHS Supplies, Leeds, UK) at room temperature, before being transferred to individual pots containing 100 ml hypotonic buffer (10 mM tris, 2.7 mM EDTA [Fisher Scientific], 10 KIU ml^−1^ aprotinin [NHS Supplies, Leeds, UK]), and incubated at 37 °C. After 24 h the solution was changed to hypotonic buffer containing the ionic surfactant sodium dodecyl sulphate (0.1%; w/v SDS [Sigma], 10 mM tris, 2.7 mM EDTA [Fisher Scientific], 10 KIU ml^−1^ aprotinin [NHS Supplies, Leeds, UK]) for 24 h. This was repeated twice. After the final hypotonic SDS wash, the tendons were washed in PBS containing 10 KIU ml^−1^ aprotinin (NHS Supplies, Leeds, UK) three times; 30 min, 70 h and a further 30 min. Each tendon was then incubated in 60 ml of nuclease solution (50 mM tris buffer, 1 mM MgCl_2_·6H_2_O [Fisher Scientific], 1 U ml^−1^ Benzonase [Merck]) at 37 °C with agitation at 80 rpm three times for 2 h each. Next, the tendons were washed three times in PBS containing EDTA (2.7 mM EDTA [Fisher Scientific]) before an overnight wash in hypertonic buffer (50 mM tris, 1.5 M sodium chloride [Fisher Scientific]). After three 30 min PBS washes, samples were sterilised using PAA (0.1%; v/v [Sigma]) for 3 h. A further 3×30 min, 2×60 h, and 1×120 h PBS washes completed the process.

Hence, four groups were investigated in Study 1:

• Native (untreated)
• DC1
• DC1+ACE
• DC1+CM.

DC1: decellularisation process without any fat reduction treatment, ACE: acetone treatment, CM: chloroform–methanol treatment, + denotes ‘with’.

**Fig. 1:**
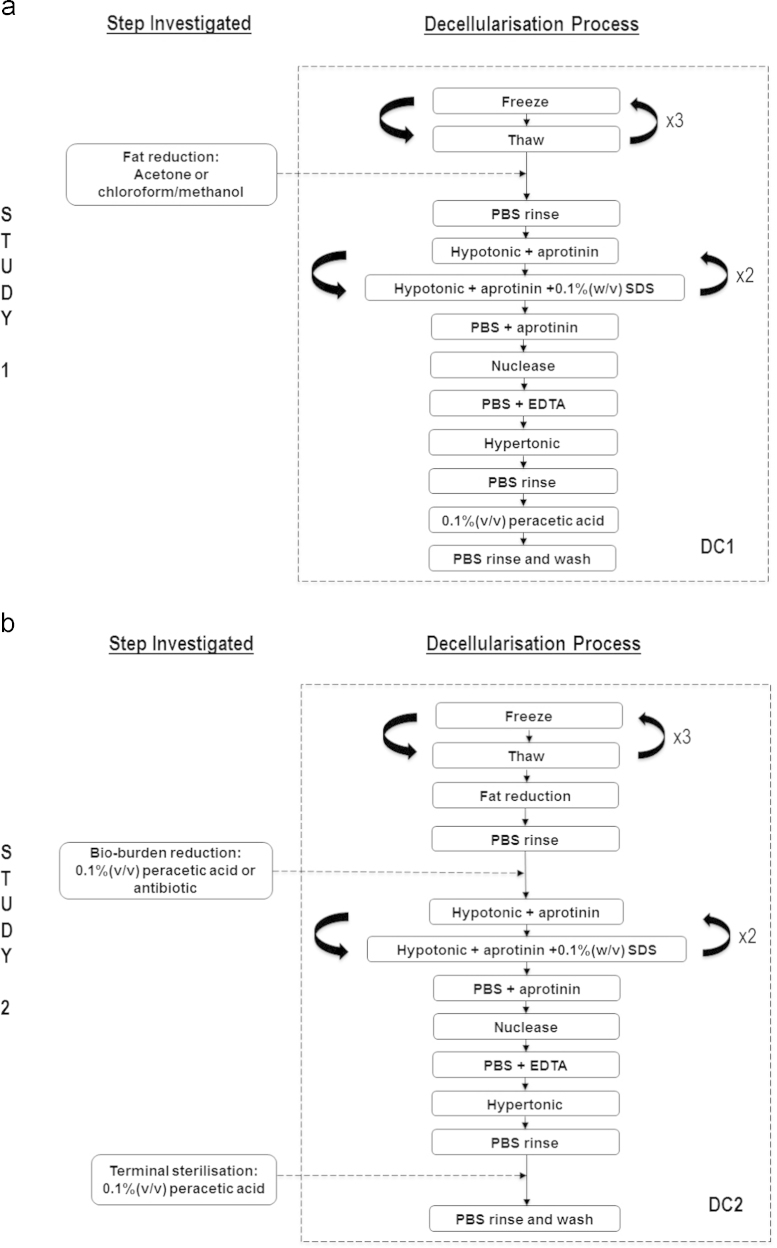
(a) Study 1: the inclusion of acetone or chloroform–methanol as a fat reduction step, (b) Study 2: the inclusion of peracetic acid or antibiotics as a bio-burden reduction step and how these performed with/without peracetic acid as a terminal sterilant.

The steps investigated and the processes involved are shown in Fig. 1(a).

#### 2.2.2 Study 2: Sterilisation study

The decellularisation procedure for Study 2 was as described for Study 1 but with the acetone treatment included as a fat reduction step. The following changes were then subsequently introduced. A 3 h bioburden reduction step was included immediately after the acetone treatment using either peracetic acid (0.1%; v/v [Sigma]) or an antibiotic solution (PBS containing 0.05 mg ml^−1^ vancomycin hydrochloride, 0.5 mg ml^−1^ gentamicin sulphate, 0.2 mg ml^−1^ polymyxin [all from Sigma]), both at room temperature. In addition, each bioburden reduction step was investigated with and without the terminal PAA treatment described in Study 1. This was to determine any interacting effects it may have with the bioburden steps on the mechanical properties of the tissue.

Hence, seven groups were investigated in Study 2:

• Native (untreated)
• DC2+TPAA
• DC2−TPAA
• DC2+PAAbio+TPAA
• DC2+PAAbio−TPAA
• DC2+ABbio+TPAA
• DC2+ABbio−TPAA.

DC2: decellularisation process with acetone permanently included (i.e. DC2=DC1+ACE), TPAA: terminal peracetic acid treatment, PAAbio: peracetic acid bioburden treatment, ABbio: antibiotic bioburden treatment. + and − denote ‘with’ and ‘without’ respectively.

The steps investigated and the processes involved are shown in Fig. 1(b).

### 2.3 Biomechanical testing

#### 2.3.1 Specimen preparation

For each group investigated, pSFT’s were removed from storage and immersed in dry ice to aid processing them into dumbbell shapes with a working cross-sectional area of 3.5×5 mm and gauge length of 30 mm. All specimens were then wrapped in PBS soaked filter paper and allowed to thaw and equilibrate at room temperature for at least 2 h prior to mechanical testing.

#### 2.3.2 Stress relaxation testing

**Fig. 2:**
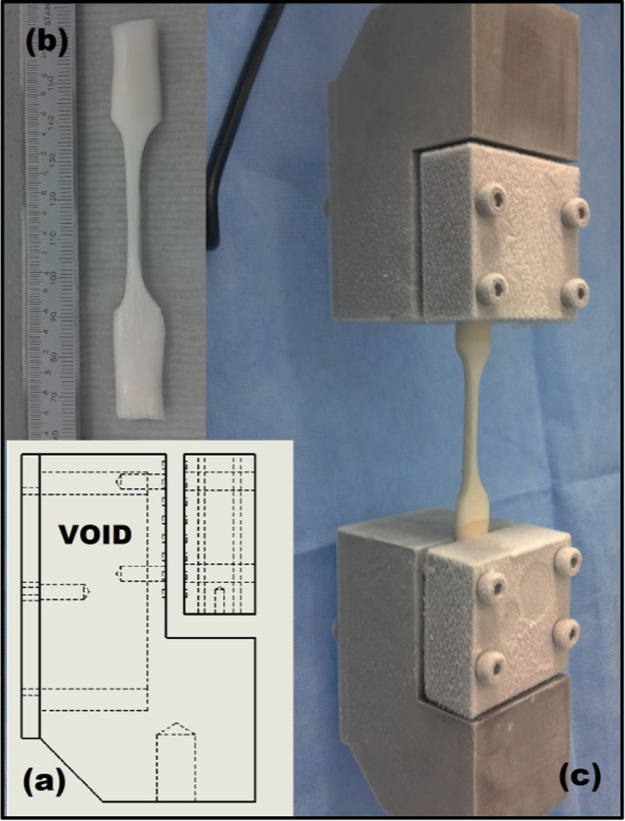
Experimental set-up for stress relaxation testing: (a) schematic of tissue grips including void for dry ice, (b) a decellularised specimen is processed to a dumbbell shape and (c) specimen is mounted in the grips and subjected to testing.

Specimens were mounted via bespoke grips (Fig. 2) to an Instron 3365 (Instron, Bucks, UK) materials testing machine equipped with a 500 N load cell. The grips were manufactured to utilise dry ice to apply the long established principles of ‘cryo-grips’ (Riemersa and Schamhardt, 1982). A probe was used to measure the temperature of the specimens at the gauge length, to ensure they had remained at room temperature and had not been adversely affected by the freezing action of the grips.

Once secured in position, specimens were tensioned to a pre-load of 0.5 N, followed by 10× preconditioning cycles between 0 and 5% strain at a rate of 15 mm min^−1^. A ramp and hold cycle was then applied consisting of a ramp phase at a rate of 30 mm min^−1^ until a stress of 5 MPa was achieved. This stress was deemed physiologically relevant based on studies determining the tensile load experienced by the ACL in-vivo (Fleming and Beynnon, 2004; Hosseini et al., 2011; Shelburne et al., 2004) and its cross-sectional area (Hashemi et al., 2005). The specimens were then held at the strain developed at the end of the ramp phase for a period of 300 s whilst stress relaxation occurred. Data was recorded at a frequency of 10 Hz. Stress (*σ*) was calculated by the dividing the force recorded by the load cell by the working cross-sectional area of the specimen, whereas strain (*ε*) was determined by dividing the crosshead displacement by the gauge length of the specimen.

The relaxation modulus (*E*(*t*)) was calculated from the experimental data using the following relationship:

$$E\left(t\right)=\frac{\sigma (t)}{\varepsilon }$$

and fitted (*r*^2^>0.96) to a modified Maxwell–Wiechert model using the non-linear least squares method (Jimenez Rios et al., 2007):

$$E\left(t\right)=E_{0}+\frac{1}{t_{0}}\sum_{i=1}^{n}E_{i}\tau_{i}\times \exp^{\left(-(t/\tau_{1})\right)}\left(\exp^{\left(t_{0}/\tau_{i}\right)}-1\right)$$

The modification performed to the model accounted for any stress relaxation that may have occurred during the ramp phase (*0*≤*t*≥*t*_0_). The simplest form of the model consists of two Maxwell elements in parallel with a single spring (i.e. *n*=2). *E*_0_ is the time-independent elastic modulus of the single spring, whereas *E*_*i*_ and *τ*_*i*_ represent the time-dependent elastic modulus and relaxation time respectively of the Maxwell elements

#### 2.3.3 Strength testing

After stress relaxation testing was performed, all specimens were wrapped in PBS soaked filter paper and allowed to equilibrate at room temperature for at least 2 h prior to strength testing. Specimens were mounted to an Instron 3366 (Instron, Bucks, UK) materials testing machine equipped with a 1000 N load cell. A pre-load of 0.5 N was applied, followed by 10× preconditioning cycles between 0 and 5% strain at a rate of 15 mm min^−1^ and a ramp to failure at a rate of 30 mm min^−1^. Failure was defined as mid-substance rupture. Data was recorded at a frequency of 10 Hz.

For each specimen, the following Gaussian function was fitted (*r*^2^>0.99) to the stress-strain data up to the failure point using non-linear least squares regression:

$$\sigma \left(\varepsilon \right)=\sum_{i=1}^{3}a_{i}\times \exp^{\left(-{\left(\varepsilon -b_{i}/c_{i}\right)}^{2}\right)}$$

where *a*_*i*_, *b*_*i*_, and *c*_*i*_ are constants to be determined.

**Fig. 3:**
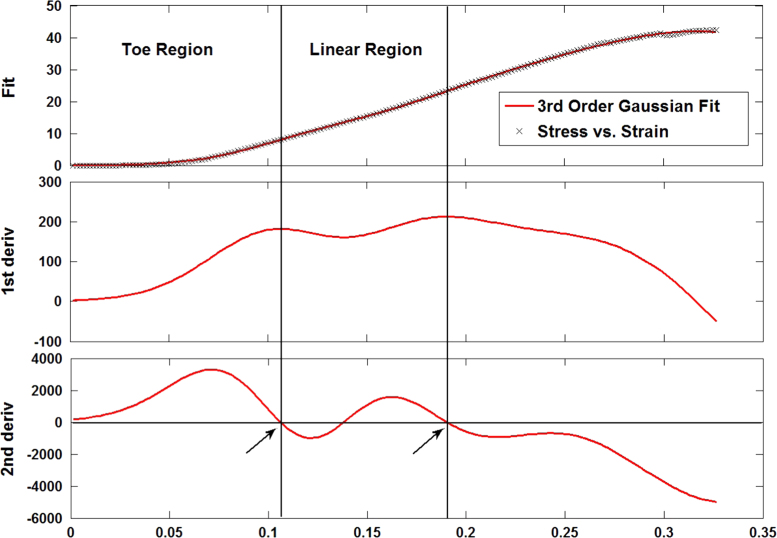
The experimental stress–strain data was fitted to a 3rd order Gaussian function from which the first and second derivatives were calculated. The first location at which the second derivative was zero was interpreted as the transition point from the toe-region into the linear-region. The third location at which the second derivative was zero was interpreted to mark the end of the linear-region.

This was differentiated twice and the locations at which the 2nd order differential was found to be zero were determined. The first of these was interpreted to mark the transition from the toe-region of the curve into the linear-region and the third marked the end of the linear-region. The corresponding strain values for these points were noted and used to distinguish between the experimental data for the toe-region and the linear-region (indicated with arrows in Fig. 3). The transition point between both regions (*ε*_*T*_, *σ*_*T*_) comprised of the transition strain (*ε*_*T*_) and transition stress (*σ*_*T*_).

Stress–strain data in the toe-region was fitted (*r*^2^>0.97) to the following exponential function:

$$\sigma \left(\varepsilon \right)=A\times \exp^{\left(B\times \varepsilon -1\right)}$$

where *A* is a constant of magnitude and *B* describes the sensitivity of *σ*(*ε*) to increasing strain.

This can be differentiated with respect to *ε* to yield the tangent modulus describing the toe-region:

$$\frac{d\sigma (\varepsilon )}{d\varepsilon }=AB\times \exp^{\left(B\times \varepsilon \right)}$$

hence, *AB* represents the zero-strain tangent modulus.

Stress–strain data in the linear-region was fitted to a linear function, the slope of which was deemed to be the Young’s modulus of the collage phase of the tissues (*E*). Finally, the ultimate tensile strength (*UTS*) and failure strain (*ε*_*FAIL*_) were determined at the point of failure.

### 2.4 Statistical analyses

Statistical variances between groups were determined using a one-way analysis of variance (ANOVA). Tukey’s significant difference test was used for post-hoc evaluation. A *P*-value of <0.05 was considered to be statistically significant.

## 3 Results

### 3.1 Study 1: Fat reduction study

The stress relaxation testing results (Table 1) showed significant differences for the parameters *E*_0_ and *E*_1_ for all decellularised groups compared to the native control. For the parameters *E*_2_ and *τ*_2_, the use of acetone to reduce the fat content appeared to have a positive effect on the properties of the pSFT, as no significant difference was found between DC1+ACE and the native specimens.

**Fig. 4:**
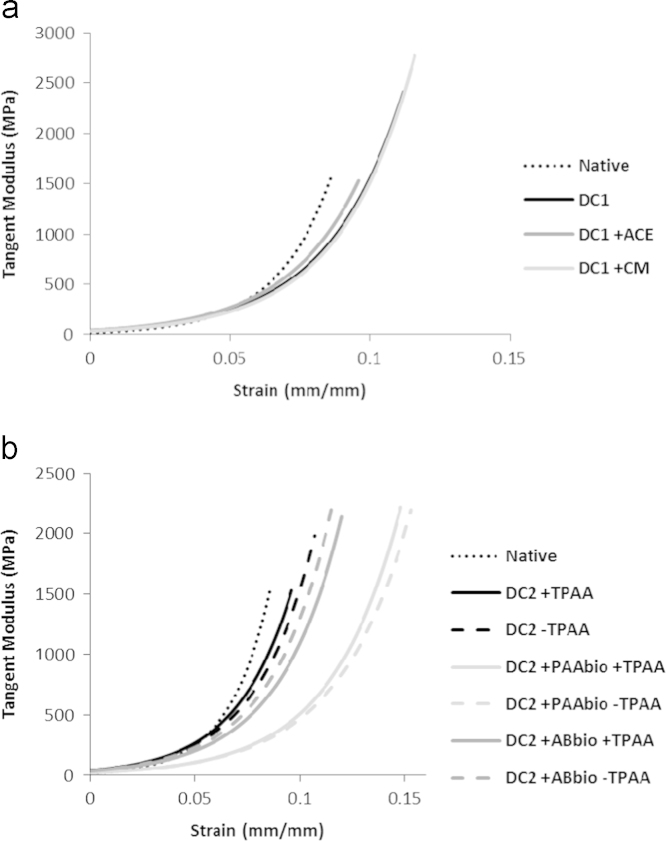
The mean tangent modulus (constructed using the mean values for parameters *A* and *B*) during the early phase of loading in the toe-region for the groups in (a) Study 1 and (b) Study 2. In each case the profile terminates at the transition strain (*ε*_*T*_). ACE: acetone treatment, CM: chloroform–methanol treatment, TPAA: terminal peracetic acid treatment, PAAbio: peracetic acid bioburden treatment, ABbio: antibiotic bioburden treatment.

The results from the strength testing (Table 2) showed no significant variation between the groups for the clasical material paramters of *UTS*, *E* and *ε*_*FAIL*_. Differences were, however, found to occur in the early phase of loading. Native tissue had a significantly lower transition strain (*ε*_*T*_) compared to the tissue that had been decellularised and treated with chloroform–methanol, although the transition stress (*σ*_*T*_) had not changed. The parameter *A* was significantly lower for native tissue compared to all the other decellularised tissues. No differences were found for the zero-strain tangent modulus (*AB*). When the mean tangent modulus in the toe-region was calculated for these groups, DC1+ACE appeared to be closest to representing the native control (Fig. 4a).

Due to the apparent positive effect acetone had in the stress relaxation testing (*E*_2_ and *τ*_2_) and the negative effect treatment with chloroform–methanol had on the transition strain and tangent modulus in the strength testing, acetone was deemed to be the superior candidate as a fat reduction step. Hence, acetone was included as a permanent feature of the decellularisation process used in Study 2.

### 3.2 Study 2: Sterilisation study

The results of the stress relaxation testing performed in Study 2 are presented in Table 3. As was found in Study 1, significant differences were found for the parameters *E*_0_ and *E*_1_ for all decellularised groups against the native control. For *E*_0_, the groups DC2+PAAbio+TPAA and DC2+PAAbio−TPAA were significantly lower than all other decellularised groups. For *E*_1_, DC2+PAAbio−TPAA was significantly lower than DC2+TPAA, with no difference between the remaining decellularised groups. For *E*_2_, both DC2+PAAbio+TPAA and DC2+PAAbio−TPAA were significantly lower than DC2+TPAA, with no difference between the rest of the decellularised groups. In the case of the time constants, DC2+PAAbio+TPAA was significantly different than the native control for *τ*_1_ and both DC2+PAAbio+TPAA and DC2+PAAbio−TPAA were significantly different than the control for *τ*_2_.

The strength testing demonstrated that in most cases there were no significant differences found for *UTS*, *E* and *ε*_*FAIL*_ in Study 2 (Table 4). The only exception was that the group DC2+ABbio+TPAA was found to have a higher *UTS* than both DC2+TPAA and DC2−TPAA. Although no differences were found for the parameters *A* and *AB*, significant differences were found to occur in the remaining parameters defining the early phase of loading of the groups. The groups DC2+PAAbio−TPAA, DC2+ABbio+TPAA and DC2+ABbio−TPAA were all found to possess significantly higher transition stresses compared to the native control, although no significant differences were found between decellularised groups. For transition strain, DC2+PAAbio+TPAA and DC2+PAAbio−TPAA were significantly larger and little significance was found between decellularised groups. The dimensionless *B* parameter was found to be significantly lower in the groups DC2+PAAbio+TPAA, DC2+PAAbio−TPAA, DC2+ABbio+TPAA and DC2+ABbio−TPAA compared to native specimens, with no significant differences between decellularised groups.

When the mean tangent modulus was calculated, the inclusion of both bioburden reduction steps had an effect, shifting the profiles to the right of the graph meaning more strain was required for the specimens to enter the linear-region of their respective load curves (Fig. 4b). This appeared to be more substantial when PAA was used as the bioburden reduction agent.

## 4 Discussion

In the pursuit of decellularisation, many agents and processes are used, which may adversely affect the extra-cellular matrix (ECM) constituents and mechanical properties (Ingram et al., 2007; Tischer et al., 2010; Woods and Gratzer, 2005). Thus, the aim of this study was to mechanically characterise an acellular pSFT following different iterations of the decellularisation process in an attempt to optimise it. This was achieved via two separate studies; Study 1 investigated the use of acetone and chloroform–methanol as a fat reduction step whereas Study 2 investigated the use of PAA or antibiotics as a bioburden reduction step and their possible interactions with PAA as a terminal sterilisation step.

The stress relaxation results from Study 1 demonstrated a significant decrease in the time-independent and time-dependent moduli for all the decellularised groups (Table 1). Few differences were found in the time constants, although the most altered was found to be *τ*_2_ in the chloroform–methanol group. The cause of the reduction in *E*_1_ and *E*_2_ (the time-dependent moduli) appears relatively intuitive. The removal of the cellular material created a more porous and open extracellular matrix and a porous scaffold is highly desirable for cell seeding or infiltration of native cells (Ge et al., 2006). However, this feature also facilitated increased interstitial fluid flow and reduced viscous resistance. The viscosity can be described as the product of the time-dependent modulus and its corresponding time constant (*E*_*i*_*τ*_*i*_). Hence, the viscosity within each of the Maxwell elements of the viscoelastic model was reduced. It has previously been suggested that the steady-state viscoelastic response of tendons may be predominately caused by fluid exudation (Buckley et al., 2013). Furthermore, through stress relaxation studies of mouse tail tendon, it has been suggested that the time-dependent properties are largely due to poroelastic effects (Yin and Elliott, 2004). Thus, given this apparent mechanical dependence on fluid movement, it is likely that the reduced viscosities observed for decellularised groups in this study were due to the removal of the cellular material and consequently increased fluid flow. The reduction in *E*_0_ (the time-independent modulus) is an indicator of a reduction in the equilibrium elasticity of the decellularised tendons. Collagen crimping is a phenomenon that has been suggested to provide stiffness in low loading conditions (Hansen et al., 2002; Miller et al., 2012a, 2012b); hence, the decellularisation process may have consequently altered the crimping pattern. It has been demonstrated that there is increased extensibility with reduced fibril crimp periods in mitral valve chordae tendineae (Liao and Vesely, 2003) and a similar mechanical response has been observed at different sites in human patellar tendons in which differences in the crimp period exist (Stouffer et al., 1985). Thus, contraction in the tissue and a reduction in the crimp period due to the decellularisation process may explain the reduced equilibrium elasticity via increased extensibility.

This was demonstrated further in the analysis of the toe-region in the strength testing. The mean tangent modulus profiles indicated that decellularisation had decreased the initial stiffness of the tissues, with more strain required to match equivalent stress levels in the native control group (Fig. 4a). Furthermore, more strain was necessary for the specimens to reach the linear-region of the load curve, a region synonymous with the full alignment and extension of the collagen fibres (Allen et al., 1999; Wang, 2006). This trend was also observed in portions of the inferior glenohumeral ligament, leading the authors to suggest that the anterior pouch is highly crimped and requires greater strain before reaching similar levels of stress in the superior and posterior portions (Bigliani et al., 1992). In our study, although changes were observed in the toe-region of decellularised specimens, they were not witnessed elsewhere, as the *UTS*, *E* and *ε*_*FAIL*_ remained comparable to native tissues. Bigliani et al. (1992) suggested that if the amount and density of collagen fibers in regions of ligament remain consistent, then ultimate stress values remain similar, but that the stress–strain profile can vary based on the micro-organisation of the fibers. Classical material properties such as *UTS* have previously been used to examine decellularisaion effects on tissues (Ingram et al., 2007; Pridgen et al., 2011; Woods and Gratzer, 2005). Hence, it is now apparent that a broader examination of the stress–strain curves, such as that reported here, is required for complete characterisation. Study 1 investigated the mechanical effects when either acetone or chloroform–methanol was introduced to aid fat removal. Due to its superior performance over chloroform–methanol in aspects of both the stress relaxation testing (*E*_2_ and *τ*_2_) and the tangent modulus in the strength testing, acetone was chosen to be included. However, there are other reasons to promote the use of acetone. It is a universially accepted solvent and is washed from specimens more easily than chloroform–methanol as it is miscible in water and chloroform is not.

In Study 2, the stress relaxation results exhibited a similar trend to Study 1 with significant differences seen for the decellularised groups against the native group for most of the moduli determined. As the majority of the decellularisation process had remained the same, this could be attributed to the same effects suggested for Study 1, namely a changing of the collagen crimping pattern altering *E*_0_ and increased interstital fluid flow altering *E*_1_ and *E*_2_. Amongst the decellularised groups, the largest reductions appeared to have occurred when PAA was employed as a bioburden reduction step. This was a feature also discovered when the toe-region was examined in the strength testing. The most severe deviations from the profile of the native tangent modulus were found to occur when PAA was used for bioburden reduction, regardless of whether PAA was used again as a terminal sterilant or not. This is shown in Fig. 4b, with significant shifting to the right of the profiles for DC2+PAAbio+TPAA and DC2+PAAbio−TPAA indicative of a substantially reduced stiffness within the tissues. Hence, it appears that a PAA treatment step early in the decellularisation process adversely affected the toe-region mechanics, much more so than with the use of antibiotics. Acetic acid has been shown to cause a reduction in the mechanical properties of bovine pericardium (Dong et al., 2009), but it is understood that PAA has little effect on ECM’s (Crapo et al., 2011; Gilbert et al., 2006). Hence, it is unclear why use of PAA in the initial steps of the process had such an effect. The most likey explanation is that it had an interaction with subsequent treatment steps, a cascade that resulted in ECM alteration. The decellularisation processes had largely no effect on the *UTS*, *E* and *ε*_*FAIL*_ in Study 2, again emulating the trends observed in the first study.

There are limitations in this study, however. First, the strain experienced by the specimens was measured using the crosshead displacement. This assumed that preferential extension occurred at the smaller cross-sectioned gauge length and that this equates to the crosshead displacement. Strain measurement was attempted using a digital video extensometer, however difficulty was encountered isolating and contrasting the reference markers on wet tissue against the local background. A second limitation was that testing was not performed using a saline water bath. Such a system ensures specimens remain fully hydrated and at physiological temperature and pH throughout testing. However, this would have precluded the use of cyro-grips, which provided the dual benefits of secure fixation and ensuring mid-substance failure occurred on each occasion.

In conclusion, this study describes characterisation work in the development of an acellular, biocompatible graft for reconstruction of the ACL. Acetone was found to be a more effective solvent as a fat reduction step than chloroform–methanol, whereas antibiotics were preferable to PAA as a bioburden reduction step. The use of a freely sourced xenogenic biomaterial eliminates issues such as donor site morbidity with autografts and limited supply with allografts. More fundamentally, this study presents essential new knowledge of the effect of different elements of decellularisation bioprocesses, which can aid in the development of future bioprocesses and decellularised scaffolds for soft tissue repair.

## Conflict of interest statement

J. Fisher and E. Ingham are paid consultants to and shareholders of Tissue Regenix Group plc.

## Figures and Tables

**Table 1:** Study 1: results from stress relaxation testing (mean ±95% CI). Superscripts indicate significance—groups that do not share the same letter are significantly different (1-way ANOVA with Tukey post-hoc analysis). DC1: decellularisation process without any fat reduction treatment, ACE: acetone treatment, CM: chloroform–methanol treatment.

Group	*E*_0_ (MPa)	*E*_1_ (MPa)	*E*_2_ (MPa)	*τ*_1_ (s)	*τ*_2_ (s)
Native	71.67±7.37^**(a)**^	12.77±1.11^**(a)**^	11.98±3.87^**(a)**^	4.57±0.63^**(a)**^	147.03±11.73^**(a)**^
DC1	40.71±2.64^**(b)**^	6.06±1.12^**(b)**^	6.89±1.70^**(b)**^	5.47±0.50^**(a)**^	122.10±10.32^**(b)**^
DC1+ACE	42.14±7.32^**(b)**^	6.63±1.68^**(b)**^	7.33±1.89^**(a)**^	5.04±0.89^**(a)**^	133.90±13.88^**(a)**^
DC1+CM	41.75±3.82^**(b)**^	5.68±1.39^**(b)**^	6.22±1.46^**(b)**^	5.97±0.47^**(b)**^	114.92±10.12^**(b)**^

**Table 2:** Study 1: results from strength testing (mean ±95% CI). Superscripts indicate significance—groups that do not share the same letter are significantly different (1-way ANOVA with Tukey post-hoc analysis). DC1: decellularisation process without any fat reduction treatment, ACE: acetone treatment, CM: chloroform–methanol treatment.

Group	*σ*_*T*_ (MPa)	*ε*_*T*_ (mm mm^−1^)	*A* (MPa)	*B* (dim’less)	*AB* (MPa)	*UTS* (MPa)	*E* (MPa)	*ε*_*FAIL*_ (mm mm^−1^)
Native	9.83±1.68^**(a)**^	0.086±0.001^**(a)**^	0.40±0.12^**(a)**^	50.63±3.19^**(a)**^	19.97±5.27^**(a)**^	52.22±4.62^**(a)**^	235.15±31.56^**(a)**^	0.315±0.028^**(a)**^
DC1	11.30±1.96^**(a)**^	0.112±0.018^**(a, b)**^	1.05±0.25^**(b)**^	36.81±7.39^**(a)**^	39.08±16.01^**(a)**^	40.83±6.48^**(a)**^	202.04±22.41^**(a)**^	0.297±0.023^**(a)**^
DC1+ACE	14.22±4.07^**(a)**^	0.096±0.005^**(a, b)**^	1.11±0.29^**(b)**^	37.77±5.54^**(a)**^	40.72±5.68^**(a)**^	46.41±7.60^**(a)**^	211.29±24.89^**(a)**^	0.297±0.023^**(a)**^
DC1+CM	12.30±2.61^**(a)**^	0.116±0.022^**(b)**^	0.93±0.27^**(b)**^	37.81±10.96^**(a)**^	34.54±14.30^**(a)**^	39.01±8.10^**(a)**^	200.67±34.63^**(a)**^	0.280±0.023^**(a)**^

**Table 3:** Study 2: results from stress relaxation testing (mean ±95% CI). Superscripts indicate significance—groups that do not share the same letter are significantly different (1-way ANOVA with Tukey post-hoc analysis). DC2: decellularisation process with acetone permanently included, TPAA: terminal peracetic acid treatment, PAAbio: peracetic acid bioburden treatment, ABbio: antibiotic bioburden treatment.

Group	*E*_0_ (MPa)	*E*_1_ (MPa)	*E*_2_ (MPa)	*τ*_1_ (s)	*τ*_2_ (s)
Native	71.67±7.37^**(a)**^	12.77±1.11^**(a)**^	11.98±3.87^**(a)**^	4.57±0.63^**(a)**^	147.03±11.73^**(a)**^
DC2+TPAA	42.14±7.32^**(b, c)**^	6.63±1.68^**(b)**^	7.33±1.89^**(a, b)**^	5.04±0.89^**(a, b)**^	133.90±13.88^**(a, b)**^
DC2−TPAA	55.66±4.48^**(b)**^	5.67±1.08^**(b, c)**^	4.09±1.27^**(b, c)**^	4.82±0.92^**(a, b)**^	124.42±17.97^**(a, b)**^
DC2+PAAbio+TPAA	30.22±3.84^**(d)**^	3.86±1.79^**(b, c)**^	2.75±0.88^**(c)**^	5.95±0.79^**(b)**^	112.20±12.75^**(b)**^
DC2+PAAbio−TPAA	29.63±6.97^**(d)**^	3.44±1.02^**(c)**^	2.26±0.36^**(c)**^	6.01±0.42^**(a, b)**^	111.06±9.90^**(b)**^
DC2+ABbio+TPAA	48.45±6.72^**(b, c)**^	6.26±1.45^**(b, c)**^	3.94±0.72^**(b, c)**^	6.64±1.49^**(a, b)**^	133.32±17.96^**(a, b)**^
DC2+ABbio−TPAA	41.82±4.00^**(c, d)**^	5.02±0.78^**(b, c)**^	3.76±0.51^**(b, c)**^	5.87±0.33^**(a, b)**^	104.99±10.37^**(b)**^

**Table 4:** Study 2: results from strength testing (mean ±95% CI). Superscripts indicate significance—groups that do not share the same letter are significantly different (1-way ANOVA with Tukey post-hoc analysis). DC2: decellularisation process with acetone permanently included, TPAA: terminal peracetic acid treatment, PAAbio: peracetic acid bioburden treatment, ABbio: antibiotic bioburden treatment.

Group	*σ*_*T*_ (MPa)	*ε*_*T*_ (mm mm^−1^)	*A* (MPa)	*B* (dim’less)	*AB* (MPa)	*UTS* (MPa)	*E* (MPa)	*ε*_*FAIL*_ (mm mm^−1^)
Native	9.83±1.68^**(a)**^	0.086±0.001^**(a)**^	0.40±0.12^**(a)**^	50.63±3.19^**(a)**^	19.97±5.27^**(a)**^	52.22±4.62^**(a, b)**^	235.15±31.56^**(a)**^	0.315±0.028^**(a)**^
DC2+TPAA	14.22±4.07^**(a, b)**^	0.096±0.005^**(a)**^	1.11±0.29^**(a)**^	37.77±5.54^**(a, b)**^	40.72±5.68^**(a)**^	46.41±7.60^**(b)**^	211.29±24.89^**(a)**^	0.297±0.023^**(a)**^
DC2−TPAA	15.72±4.12^**(a, b)**^	0.107±0.016^**(a, b)**^	1.28±0.57^**(a)**^	36.06±8.24^**(a, b)**^	41.80±12.49^**(a)**^	45.67±1.06^**(b)**^	232.89±15.33^**(a)**^	0.276±0.013^**(a)**^
DC2+PAAbio+TPAA	16.94±3.40^**(a, b)**^	0.148±0.031^**(b, c)**^	0.94±0.39^**(a)**^	30.68±10.33^**(b)**^	23.67±4.98^**(a)**^	51.12±8.12^**(a, b)**^	240.92±38.18^**(a)**^	0.312±0.030^**(a)**^
DC2+PAAbio−TPAA	18.21±1.48^**(b)**^	0.153±0.024^**(c)**^	0.91±0.66^**(a)**^	29.12±3.05^**(b)**^	25.26±16.70^**(a)**^	49.94±5.10^**(a, b)**^	266.27±43.65^**(a)**^	0.313±0.023^**(a)**^
DC2+ABbio+TPAA	18.19±3.58^**(b)**^	0.120±0.021^**(a, b, c)**^	1.24±0.53^**(a)**^	33.53±5.86^**(b)**^	38.26±11.26^**(a)**^	65.18±9.70^**(a)**^	317.13±43.78^**(a)**^	0.292±0.028^**(a)**^
DC2+ABbio−TPAA	18.53±3.29^**(b)**^	0.116±0.021^**(a, b, c)**^	1.26±0.53^**(a)**^	34.77±5.78^**(b)**^	40.14±10.97^**(a)**^	61.52±4.53^**(a, b)**^	296.77±25.59^**(a)**^	0.267±0.018^**(a)**^
